# Systematic Characterization of the Group 2 House Dust Mite Allergen in *Dermatophagoides microceras*

**DOI:** 10.3389/fcimb.2021.793559

**Published:** 2022-01-17

**Authors:** Rei-Hsing Hu, Chia-Ta Wu, Ting-Shuan Wu, Feng-Yih Yu, Jiunn-Liang Ko, Ko-Huang Lue, Yu-Fan Liu

**Affiliations:** ^1^ Department of Biomedical Sciences, Chung Shan Medical University, Taichung, Taiwan; ^2^ Institute of Medicine, Chung Shan Medical University, Taichung, Taiwan; ^3^ Department of Emergency Medicine, Changhua Christian Hospital, Changhua, Taiwan; ^4^ Graduate Institute of Toxicology, College of Medicine, National Taiwan University, Taipei, Taiwan; ^5^ Division of Allergy, Department of Pediatrics, Chung Shan Medical University Hospital, Taichung, Taiwan

**Keywords:** allergen, HDMs, asthma, OMICS, FIP-*fve*, Der m 2, *Dermatophagoides microceras*

## Abstract

**Background:**

Allergic asthma, a chronic airway inflammatory disease, is a critical public health problem. Indoor house dust mites (HDMs) could cause allergic asthma. The prevalence of sensitization to *Dermatophagoides microceras* (*Der m*) was approximately 80% and is related to the immunoglobulin E crossing-reactivity of mites belonging to the same genus, *Dermatophagoides pteronyssinus* (*Der p*) and *Dermatophagoides farina* (*Der f*). However, studies on *Der m* are scant.

**Methods:**

We used integrated OMICs approaches to identify and characterize the group 2 mite allergen-like protein in *Der m* (*Der m 2*). We established a *Der m 2*-induced allergic asthma mouse model and treated the mice with a fungal immunomodulatory protein (FIP-*fve*) isolated from *Flammulina veluptipes* to evaluate the allergenicity of *Der m 2* and the immunomodulatory effects of FIP-*fve*.

**Results:**

By performing *de novo* draft genome assembly and comparative genome analysis, we identified the putative 144-amino acid *Der m 2 in silico* and further confirmed its existence through liquid chromatography–tandem mass spectrometry. *Der m 2* is a lipopolysaccharides (LPS)-binding protein. Thus, we examined the LPS-binding activity of recombinant *Der m 2* by performing molecular docking analysis, co-immunoprecipitation (Co-IP), and a pull-down assay. *Der m 2* elicited the production of pro-inflammatory cytokines, interleukin (IL)-6, and IL-8 in BEAS-2B cells, a human bronchial epithelial cell line, and induced airway hyperresponsiveness in mice. Furthermore, in mice sensitized with *Der m 2*, the administration of FIP-*fve* in either the earlier stage or the late stage, FIP-*fve* alleviated allergic asthma by moderating airway inflammation and remodeling.

**Conclusions:**

*Der m 2* induced inflammatory responses in cell and mouse models. FIP-*fve* alleviated inflammation in *Der m 2*-induced asthma in mice by exerting an immunomodulatory effect.

## Introduction

Asthma is a major noncommunicable disease among preschool children and is characterized by airway inflammation, goblet cell metaplasia, and airway remodeling. In the United States, more than 339 million people have asthma (Disease et al., 2017), and the medical expenditure for asthma was USD 81.9 billion in 2013 (Nurmagambetov et al., 2018). Risk factors for asthma commonly present in the environment include outdoor pollen, molds, indoor mites, and air pollution. House dust mites (HDMs) are the major risk factors for asthma in tropical urban regions because these allergens were easily recognized by human immunoglobulin E (IgE) (Andiappan et al., 2014). Children with atopic allergy in central Taiwan had higher sensitivity to HDMs than other indoor allergens such as pet dander, cockroach, and fungi. The major HDM species—including *Dermatophagoides pteronyssinus* (*Der p*), *Dermatophagoides farina* (*Der f*), *Dermatophagoides microceras* (*Der m*), and *Blomia tropicalis* (*Blo t*)—could produce allergens that can induce allergic immune responses and asthma (Huang et al., 2006). Although more than 30 allergens of *Der f* or *Der p* are included in the World Health Organization (WHO)/International Union of Immunological Societies (IUIS) Allergen Nomenclature database, groups 1 and 2 are the major allergens (Meyer et al., 1994; Pittner et al., 2004; Chen et al., 2012).

HDM-associated allergens induce the expression of inflammatory cytokines. The group 1 and 2 allergens of *Dermatophagoides* are the major sensitizing components (Huang et al., 2020). The structure of *Der p 2* is homologous to that of myeloid differentiation-2 (MD-2), a component of Toll-like receptor (TLR)4 signaling complex. In addition, *Der p 2* has a β-cup structure that forms a hydrophobic pocket for lipopolysaccharide (LPS) binding (Ichikawa et al., 2009; Trompette et al., 2009; Chen et al., 2018). *Der p 2* could induce the release of pro-inflammatory cytokines, including interleukin (IL)-6 and IL-8 through mitogen-activated protein kinase (MAPK) and nuclear factor-κB pathways in human bronchial epithelial cells and human B cells (Tsai et al., 2011; Pan et al., 2019). In addition, *Der f 2* could induce the expression of Th2 inflammatory-related cytokines, such as IL-4 and IL-13, in human bronchial epithelial cell and mice (Lee et al., 2018b). Allergens from HDMs can cause allergy and asthma in children. A substantially high proportion of Taiwanese children with allergy exhibited cosensitization to *Der m* as well as *Der f*, *Der p*, and *Blo t*. However, *Der m* has been rarely studied (Huang et al., 2006), and no data on *Der m 2* are available in the WHO/IUIS Allergen Nomenclature database. Moreover, the recombinant proteins of group 2 allergens of Dermatophagoides caused similar sensitizing responses to their native proteins (Jin et al., 2003; Sookrung et al., 2016).

A fungal immunomodulatory protein (FIP-*fve*) was isolated from *Flammulina velutipes*, an edible mushroom called golden needle mushroom or enoki mushroom (Ko et al., 1995). FIP-*fve*, a 13-kDa glycoprotein containing 114 amino acid residues, induced interferon (IFN)-γ production in human peripheral blood mononuclear cells (PBMCs) (Wang et al., 2004). In addition, FIP-*fve* alleviated the inflammation caused by respiratory syncytial virus (RSV) and its replication, and the oral administration of FIP-*fve* could reduce the acute airway inflammation caused by *Der p* (Chang et al., 2015; Chu et al., 2017). Moreover, oral administration of FIP-*fve* could reduce ovalbumin (OVA)-induced airway inflammation and weaken the effect of TH17 cells, thus alleviating airway remodeling (Lee et al., 2018a). Furthermore, regardless of pre- or post-administration of FIP-*fve* in the acute or chronic stage, it improved asthma symptoms in mice (Wu et al., 2020).

Our previous studies have reported 79.5% prevalence of cosensitization to *Der m* among 498 children with allergy in central Taiwan (Huang et al., 2006). Furthermore, we examined *Der m 2*, one of the major allergens of *Der m* that is not yet reported in WHO/IUIS Allergen Nomenclature database. In this study, we systematically characterized *Der m 2* and its allergenicity and investigated the immunomodulatory effects of FIP-*fve* on *Der m 2*-induced asthma to verify its immunomodulatory ability on the *Der m 2*-induced asthma in mice.

## Materials and Methods

### Whole-Genome Sequencing and OMICs Analysis

*Der m* was purchased from Thermo Scientific™ ImmunoCAP™ mite allergens, and the samples were consigned to Welgene Biotech Co., Ltd. (Taiwan), for genomic DNA preparation and next-generation sequencing (NGS) with Illumina Solexa™ technology. For the genomic DNA library construction, the Agilent SureSelectXT HS Reagent Kit protocol for Illumina Multiplexed Paired-End sequencing library was applied, and the amplified adapter-ligated sample was further analyzed with Agilent D1000 Screen Tape assay on the 4200 TapeStation system. Finally, the sample was sequenced on the Illumina sequencing platform with paired-end sequencing cycles. The paired-end reads were trimmed the adaptor sequences and the reads with the quality scores lower than 20 by Trimmomatic (Bolger et al., 2014), then the remaining reads were assembled by ABySS with 31 κ-mer size on Ubuntu server after trimming and cleaning the reads to eliminate reads with low sequencing quality. The protein sequences of *Der f 2* and *Der p 2* were obtained from WHO/IUIS Allergen Nomenclature Sub-Committee website that was used to find the homologous protein of *Der m* by BLASTP algorithm.

The liquid chromatography–tandem mass spectrometry (LC-MS/MS) was consigned to Mithra Biotechnology Inc. in Taiwan. Approximately 100 mg *Der m* was dissolved in 1 ml 0.01 M phosphate buffered saline (PBS) and was sonicated with SONICS Vibra-Cell Ultrasonic Liquid Processor VCT-130. After sonication, the solution was centrifuged to eliminate the insoluble pellet. The crude extract of *Der m* was digested with trypsin and followed by LC-MS/MS survey scan. Full MS scan was performed with ranges of m/z 300–2,000, m/z 300–600, m/z 600–800, m/z 800–1,200, and m/z 1,200–2,000, and the top 10 intense ions from MS were chosen for MS/MS scan. After scan, raw data were analyzed by Proteome Discover 1.4 for Mascot database search. There were 43 proteins identified as the mite-related proteins from NCBI database, and 29 proteins were identified as the predicted for *Der m* allergens.

### Expression and Purification of Recombinant *Der m 2*

The recombinant *Der m 2* was expressed and purified according to the protocol (Lee et al., 2011). In brief, the first 17-amino acid residues, the signal peptide, were deleted and constructed into pET29a expression plasmid. The plasmid was transformed into *E. coli* BL21 (DE3) (ECOS™21; Yeastern Biotech, Taiwan) and induced by using final 0.5 mM isopropyl-D-thiogalactoside (IPTG; Sigma-Aldrich, Cat# I6758). After induction, the bacterial cells were harvested by centrifugation at 7,000 ×g for 15 min following cell lysis and purification by the cOmplete™ His-Tag Purification Resin (Roche, Cat# 5893682001) as the aforementioned protocol. The purified recombinant *Der m 2* was further purified by HiTrap™ Q HP anion exchange chromatography to remove the excess LPS.

*Der m 2* without the signal peptide was cloned into pPICZαA plasmid, and then the constructed plasmid was transformed to *Pichia pastoris* for protein expression, yM2. The recombinant *P. pastoris* was inoculated in YPD medium at 30°C overnight and then transferred after adequate overnight culture to a fresh BMMY medium. The cell culture was incubated at 30°C for 72 h, and 0.5% (v/v) methanol was added to the medium every 24 h. After incubation, the supernatant of cell culture was harvested by centrifugation and concentrated by Amicon™ Ultra-15 centrifugal filter unit. The concentrated protein solution was further purified by Ni-NTA affinity column.

### Endogenous Lipopolysaccharide Quantitation

The LPS in recombinant *Der m 2* from *E. coli* was quantitated by using Pierce™ limulus amebocyte lysate (LAL) chromogenic endotoxin quantitation kit. The different concentrations of endotoxin, 0, 1, 3.5, 7, 14, 21, 28 EU/ml, were prepared for the standard curve, and the purified M2Q was diluted 10-fold (~50 μg/ml) as unknown sample. Each standard or sample was dispensed at 50 μl to the appropriate microplate well, and the endotoxin-free water was used as the blank. As the manufacturer’s protocol, at time (T) = 0, 50 μl LAL reagent was added to each well, and then 100 μl prewarmed Chromogenic Substrate solution was added at T = 10 min. Finally, the reaction was stopped by adding 100 μl 25% acetic acid at T = 16 min, and the absorbance at 405 nm was measured on a plate reader. The endogenous LPS concentration of the purified M2Q could be calculated with the standard curve.

### Purification of FIP-*fve* Protein

FIP-*fve* was purified as the protocol described in the previous study (Chu et al., 2017). In brief, 300 g golden needle mushrooms were homogenized with 1 L homogenization buffer (5% acetic acid, 0.05 M β-mercaptoethanol) after immersing in the buffer for at least 1 h on ice. The homogenized solution was centrifuged at 9,820 ×g for 20 min at 4°C, and the supernatant was added to ammonium sulfate until saturation (around 90%) to precipitate proteins. After centrifugation, the precipitate pellet was dialyzed in dialysis buffer (10 mM sodium acetate, pH 5.4) for 48 h, and then the insoluble pellet was removed by centrifugation (12,000 ×g for 40 min at 4°C). The dialyzed protein solution was further purified with cation exchange column CM-52.

### Structural Comparison and Molecular Docking Simulation

The superimposed three-dimensional (3D) structure comparison, docking simulation, and ligand–protein interaction diagram were performed by ExPASy SwissPdbViewer, Scripps AutoDock vina (Trott and Olson, 2010), and EMBL-EBI LigPlot^+^ packages (Laskowski and Swindells, 2011), respectively. The atomic coordinates and structural data were retrieved from the RCSB Protein Data Bank database under accession codes.

### Mouse Polyclonal Anti-Der m 2 Antibody

Three epitopes in Der p 2 for IgE recognition were reported, αDpx, 7A1, and 6D6 (Derewenda et al., 2002). In these three regions, the protein sequence in αDpx recognized region of Der m 2 was much different from those of Der f 2 and Der p 2. Therefore, we designed the antigenic peptides by using the protein sequence in αDpx recognized region, from P87 to L97, to immunize the mice according to the protocol in a previous study (Yu et al., 2004). The polyclonal antibodies were harvested from the sera and used in this study.

### Pull-Down Assay

The pull-down assay was based on the protocol described in the previous study (Lee et al., 2011). In detail, 50 μg recombinant *Der m 2* with or without 100 μg LPS (Sigma-Aldrich, Cat#L5418) addition was incubated with 1 ml Ni-NTA resin overnight at 4°C on the rotating mixer. After the protein mixture flowed through the column, the column was washed with washing buffer (50 mM Tris-HCl pH 7.5, 150 mM NaCl, 10% glycerol, 10 mM imidazole), whose quantity is more than 5-fold volume of resin. Finally, the proteins were eluted with elution buffer (50 mM Tris-HCl pH 7.5, 150 mM NaCl, 10% glycerol, 250 mM imidazole) and further analyzed by Western blot with the primary antibodies, anti-LPS antibody (Abcam, Cat# ab35654), and anti-*Der m 2* antibody to confirm the binding affinity between recombinant *Der m 2* and LPS.

### Co-Immunoprecipitation

The protocol followed the manual of SureBeads™ Protein A Magnetic Beads (Bio-Rad, Cat#1614013). In brief, 5 μg mouse anti-LPS antibody was incubated with 100 μl protein A magnetic beads at 4°C for 1 h, and then the beads were washed with 1 ml Phosphate buffered saline with 0.1% Tween® 20 Detergent (PBST) three times. After washing, 70 μg recombinant *Der m 2* with or without 100 μg LPS was added and incubated overnight at 4°C on the rotating mixer. After incubation, the mixed solutions were removed and washed with PBST three times. Finally, 20 mM glycine pH 2.0 was added to elute the proteins, and then the elution was transferred to 1.5 M Tris-HCl pH 8.0 to equilibrate the pH value. The eluted protein solutions were further analyzed by Western blot with the anti-*Der m 2* antibody and the rabbit anti-mouse HRP antibody (Abcam, Cat# ab97046).

### Cell-Based Experiments

The human bronchus epithelium cells, BEAS-2B, purchased from American Type Culture Collection (ATCC number CRL-9609), were cultured in LHC-9 medium (Gibco™; Thermo Fisher Scientific Inc., USA), and the fresh media were replaced before adding the allergens for the indicated treatment time according to the protocol in the previous study (Pan et al., 2019). After treatment, the media were discarded, and then 1 ml Tri reagent^®^ (Sigma-Aldrich, Cat# T9424) was added for further RNA extraction.

### Real-Time Quantitative PCR

Total RNA was extracted by using Tri reagent^®^ (Sigma-Aldrich, USA) and followed the manual of High-Capacity cDNA Reverse Transcription Kit (Cat. #4368814, Applied Biosystems™) to synthesize the first-strand cDNA. After reverse transcription, the cDNA was used as a template, and qPCR was performed with PowerUp™ SYBR™ Green Master Mix (Cat. #A25779, Applied Biosystems™) by using ABI StepOnePlus according to a protocol described in the previous study (Wu et al., 2018). The primers for qPCR are IL-6 forward 5′-TTCGGTCCAGTTGCCTTCTC-3′, IL-6 reverse 5′-GAGGTGAGTGGCTGTCTGTG-3′, IL-8 forward 5′-CTTGTCATTGCCAGCTGTGT-3′, IL-8 reverse 5′-TGACTGTGGAGTTTTGGCTG-3′, GAPDH forward 5′-ACCAGCCCCAGCAAGAGCACAAG-3′, and GAPDH reverse 5′-TTCAAGGGGTCTACATGGCAACTG-3′.

### Animal-Based Experiments

BALB/c mice were purchased from the National Laboratory Animal Center (Taipei, Taiwan). All mice were maintained in the animal center of Chung Shan Medical University to comply with regulations of Chung Shan Medical University Institutional Animal Care and Use Committee (Animal Experiment Approval number 2298). The sensitization and physiological and biochemical inspections were performed as the protocols described in previous studies (Chu et al., 2017; Wu et al., 2020), and each group had 8 mice except the normal control group (NC) that had 5. In brief, female BALB/c (6–8 weeks old) were intraperitoneally injected with 50 μg recombinant *Der m 2* on the first 3 days and were administered intranasal allergens at days 14, 17, 21, 24, and 27. Each mouse was given 200 μg FIP-*fve* by intragastric feeding every day on the first 14 days (days 1–14) or last 14 days (days 14–27). The NC was intraperitoneally injected with normal saline plus alum, and normal saline was used for intranasal administration. At day 28, the mice were challenged with methacholine to determine their airway hyperresponsiveness (AHR) by a whole-body barometric plethysmography (Model PLY 3211; Buxco Electronic Inc., Sharon, CT), and the lung function was recorded and calculated as the enhanced pause (Penh) to be a dimensionless unit that corresponds to pulmonary resistance for the resistance value (Verheijden et al., 2014). After determination of AHR, the mice were euthanized.

### Collection of Sera and Bronchoalveolar Lavage Fluid

After the mice were euthanized, from each mouse was collected around 1 ml blood by cardiac puncture to a microcentrifuge tube, and then the tubes were centrifuged at 3,000 rpm 4°C for 10 min to harvest the serum. The mice sera were stored at -80°C until use in the specific antibody assay. The mouse lung was lavaged through the trachea with 1 ml normal saline each three times to collect the bronchoalveolar lavage fluid (BALF). The cellularity of BALF was analyzed with a hemocytometer, and the differential cells were stained with Liu’s staining after using Cytospin Centrifuge at 3,000 rpm 4°C for 10 min to collect the cells on the slides. For each slide with 100 μl BALF, the classification of cells was according to the standard morphologic criteria. The remaining BALF was centrifuged at 6,000 rpm 4°C for 5 min, and the supernatant was stored at -80°C before cytokine measurement.

### Measurement of Specific Antibodies in Serum

The *Der m 2*-specific IgE and IgG1 were detected using ELISA. In brief, the 96-well microtiter plates were coated with 100 mg/ml *Der m 2* at 4°C overnight. The plates were washed with PBST before blocking with 3% bovine serum albumin (BSA) at 37°C for 1 h. After discarding the blocking solution and washing the plates, the plates were incubated with 50 μl serial diluted mouse sera in 3% BSA at 4°C overnight. Then, the plates were incubated with optimal diluted horseradish peroxidase (HRP)-conjugated anti-mouse isotype-specific antibody (BD Pharmingen™) at 37°C for 2 h. After washing the HRP-conjugated antibody solution, the TMB substrate was added into plates at room temperature for 15 min. Finally, 0.1 N HCl was added to stop the reaction, and then the absorbance of the plates at 450 nm was measured by a microplate reader.

### Mouse Cytokine Array

The mouse BALF cytokines were analyzed with cytokine array (ARY028, Proteome Profiler Mouse XL Cytokine Array, R&D Systems), according to the manual. In brief, the samples were mixed with detecting antibody at room temperature for 1 h before adding to the array membrane. The membrane with mixed solution was incubated at 4°C on a shaker overnight. Then, the membrane was washed before adding the HRP-conjugated streptavidin at room temperature on a shaker for 30 min. After another wash, the array signals on the membrane were detected by a chemiluminescence image system, and the mean of the respective pixel densities of spots were calculated by ImageJ.

### Histology

According to a protocol described in the previous study (Chu et al., 2017; Wu et al., 2020), after the mice were euthanized, the lung tissues of mice were immediately soaked in 10% formaldehyde for fixation, and then the tissues were embedded in paraffin. The paraffin blocks were sliced, and the lung tissue slices were stained with hematoxylin and eosin (H&E) to evaluate pathological changes.

### Statistical Analysis

The values are presented as mean ± SEM. The significance of qPCR results was performed by one-way ANOVA with Tukey *post hoc* test. The mouse experiments used Kruskal–Wallis test to test the significance. *P* value <0.05 was considered statistically significant.

## Results

### Confirmation of the *Der m 2* Allergen Using Whole-Genome Sequencing and OMICs Analysis

**Figure 1:**
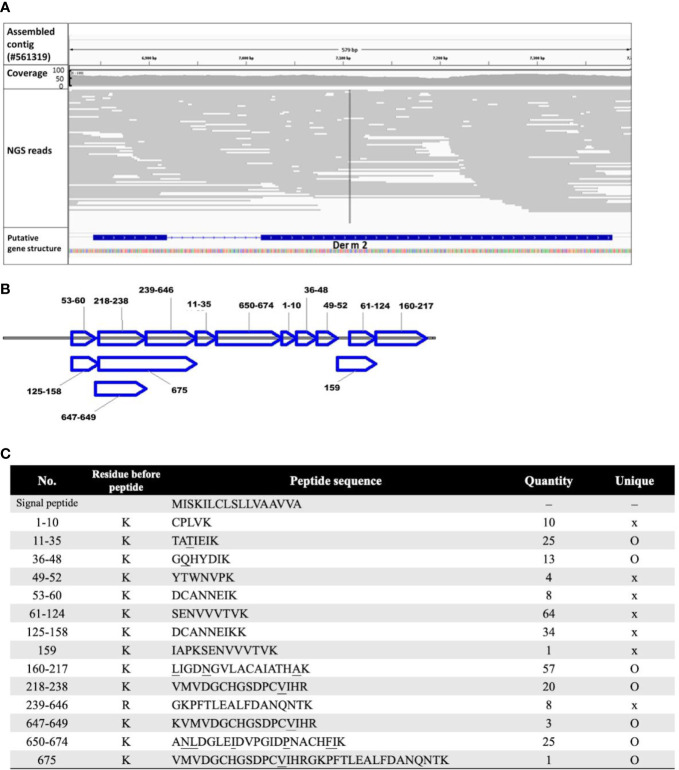
The annotated sequence of allergic *Der m 2* component. **(A)** The predicted DNA sequence of putative *Der m 2* was shown by Interactive Genomics Viewer (IGV) with the aligned NGS reads as gray lines. The first and second rows show the assembled contig sequence (config #561319; 7,784 bp) and the NGS read coverage, respectively. The third row shows the sorted NGS reads mapped to the corresponding region mismatching the assembled *Der m* genomic reference indicated by color with alpha transparency proportional to quality. The fourth row shows the predicted exon–intron structure of *Der m 2* using the sim4 package. The gene track of *Der m 2* strain oriented by arrow and the bar and line indicated the exon and intron region, respectively. **(B)** The LC-MS/MS proteomic data showed the specific fragment of deduced 146-amino acid sequence of *Der m 2*; the sequence covered 82.2%. The peptide fragments were mapped to the *Der m 2* proteins. **(C)** The signal peptide and the peptides analyzed by LC-MS/MS mass spectra of *Der m 2* and the quantity of each peptide from LC-MS/MS were listed, and the unique residues were underlined.

A total of 7,343,792 paired-end cleaned sequencing reads and approximately 1.74 billion bases of *Der m* were collected from the NGS platform. The draft genome was *de novo* assembled from the reads into 5.03 × 10^5^ contigs by using ABySS. From the genomic contigs, the putative cDNA and protein sequences of *Der m 2* were determined by GeneWise; these sequences were compared with the protein sequences, *Der f 2* (Q00855) and *Der p 2* (P49278) (Birney et al., 2004). The putative *Der m 2* gene structure consisted of two exons and one intron (**Figure 1A**) and could be translated into a 146-amino acid protein. Moreover, the first 17 amino acids of putative *Der m 2* were predicted to be a signal peptide by using SignalP 4.1 Server (**Figure 1C**).

To confirm the *Der m 2* allergen, crude extract proteins of *Der m* were analyzed through LC-MS/MS. On the basis of proteomic data, 14 unique peptides could be mapped to *Der m 2* (**Figure 1B**); their sequences were shown in **Figure 1C**. Furthermore, the DNA and protein sequences of *Der m 2* were validated using OMICs approaches, namely, WGS and proteomic analysis.

### 3D Structure Modeling and Docking Simulation for *Der m 2*

**Figure 2:**
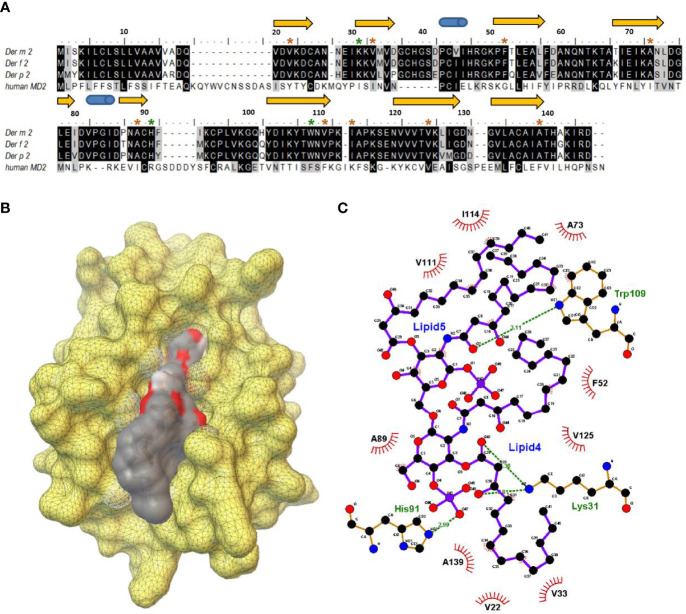
The structural characterization between the putative *Der m 2* protein and Lipid IVa complex. **(A)** The multiple-sequence alignment for the putative protein sequences of *Der m 2* with *Der f 2* (Q00855), *Der p 2* (P49278), and human MD-2 (LY96, Q9Y6Y9) based on the SWISS-MODEL “model-template” mode. The secondary structure assignment of the immunoglobulin-like β-sandwich of ML-2-related lipid recognition domain dependent on the modeling domain of *Der m 2* is shown above the sequences: arrows represented β-strands, and cylinders indicated α-helices. The color brown and green of star symbols above the sequences regarded the interesting residues of the hydrophobic contacts and hydrogen bonding interactions between *Der m 2* and Lipid IVa, respectively. **(B)** The molecular docking simulation of Lipid IVa in the hydrophobic cavity of *Der m 2* using the Scripps AutoDock vina package. The yellow color demonstrated the molecular surface of *Der m 2*. The electrostatic potential of ligand lipid IVa revealed the negative electrostatic and hydrophobic contacts in color red and gray, respectively. **(C)** Schematic representation of key residues of the *Der m 2* bound with the Lipid IVa ligand. The 2D diagram was generated with LigPlot+ that hydrogen bonds are shown as green dotted lines, while the spoked arcs represented residues making hydrophobic contacts with the ligand.

We examined the multiple-sequence alignment and superimposed structures of *Der m 2*, *Der f 2*, and *Der p 2* and observed that the protein sequences of *Der m 2* exhibited 95% similarity to those of *Der f 2* and 86% similarity to those of *Der p 2* (**Figure 2A**). Moreover, the β-cup 3D structures of *Der m 2* showed similarity to those of *Der f 2* and *Der p 2* (**Supplementary Figure S1A**). The conserved hydrophobic amino acid residues presented on β-sheets in the allergen formed a large cup-shaped cavity (**Figure 2B**). The findings of molecular docking simulation indicated that the hydrophobic cleft might assist in the binding of non-polar substrates such as Lipid IVa. Homologous comparative protein structure modeling was performed using MD-2 and *Der m 2*, and the structures of various allergies were compared (**Supplementary Figure S1B**). For instance, the hydrophobic pocket of *Der m 2* consisted of nine conserved side chains, namely, V22, V33, F52, A73, A89, V111, V114, V125, and A139, that were in hydrophobic contacts with the acyl Lipid4 and Lipid5 chains of an established ligand (**Figure 2C**). The two phosphate groups of the ligand interacting with positively charged residues exhibited four hydrogen bonds with the K31, H91, and Trp109 of *Der m 2* (**Figure 2C**).

### Molecular Function of Recombinant *Der m 2*

**Figure 3:**
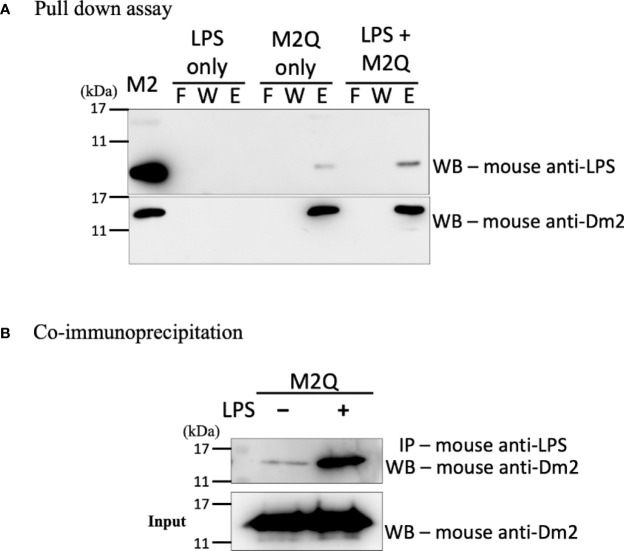
The interaction between recombinant *Der m 2* protein and LPS. **(A)** The recombinant truncated *Der m 2* protein after purifying with Q column (M2Q) was immobilized on Ni-NTA resin and then incubated with LPS for pull-down assay. The fractions of the pull-down assay were further analyzed by Western blot by using anti-LPS antibodies. The endogenous LPS signal is more apparent in the elution when LPS is present, and the signal in the elution of M2Q only should be the endogenous LPS. The Western blot using anti-*Der m 2* antibodies is to confirm the M2Q proper binding and quantity. The purified recombinant Der m 2 before Q column (M2) was shown in the first lane. F, flow through; W, wash; E, elution. **(B)** The co-immunoprecipitation (Co-IP) assay used anti-LPS antibody to precipitate LPS and further analyzed by Western blot to detect the M2Q presence. The M2Q signal is dramatically increased when LPS is present.

To examine whether the *Der m 2* allergen alone can bind to LPS, we performed anionic-exchange chromatography to remove the excess LPS of recombinant *Der m 2* purified from *Escherichia coli* (Magalhaes et al., 2007), and then two experiments were performed to evaluate binding with LPS. First, we immobilized the recombinant *Der m 2* obtained from *E. coli* (M2Q) on Ni-NTA resin, and the mixture was incubated with or without LPS. Subsequently, the flowing through, washing, and eluting steps were performed, and the solution obtained in each step was subjected to Western blot analysis. The eluent of M2Q without LPS exhibited an endogenous LPS signal, whereas that of M2Q with LPS exhibited a more prominent signal (**Figure 3A**). We performed another co-immunoprecipitation (Co-IP) assay by using the immobilized anti-LPS antibody and protein A magnetic beads to examine the binding of the antibody with M2Q with or without additional LPS. The results revealed that a small amount of M2Q without additional LPS bound to the anti-LPS antibody because of the presence of endogenous LPS in M2Q; however, M2Q with additional LPS exhibited a more prominent signal (**Figure 3B**).

### Recombinant *Der m 2* Protein Induced Proinflammatory Cytokine Expression in Human BEAS-2B Cells

**Figure 4:**
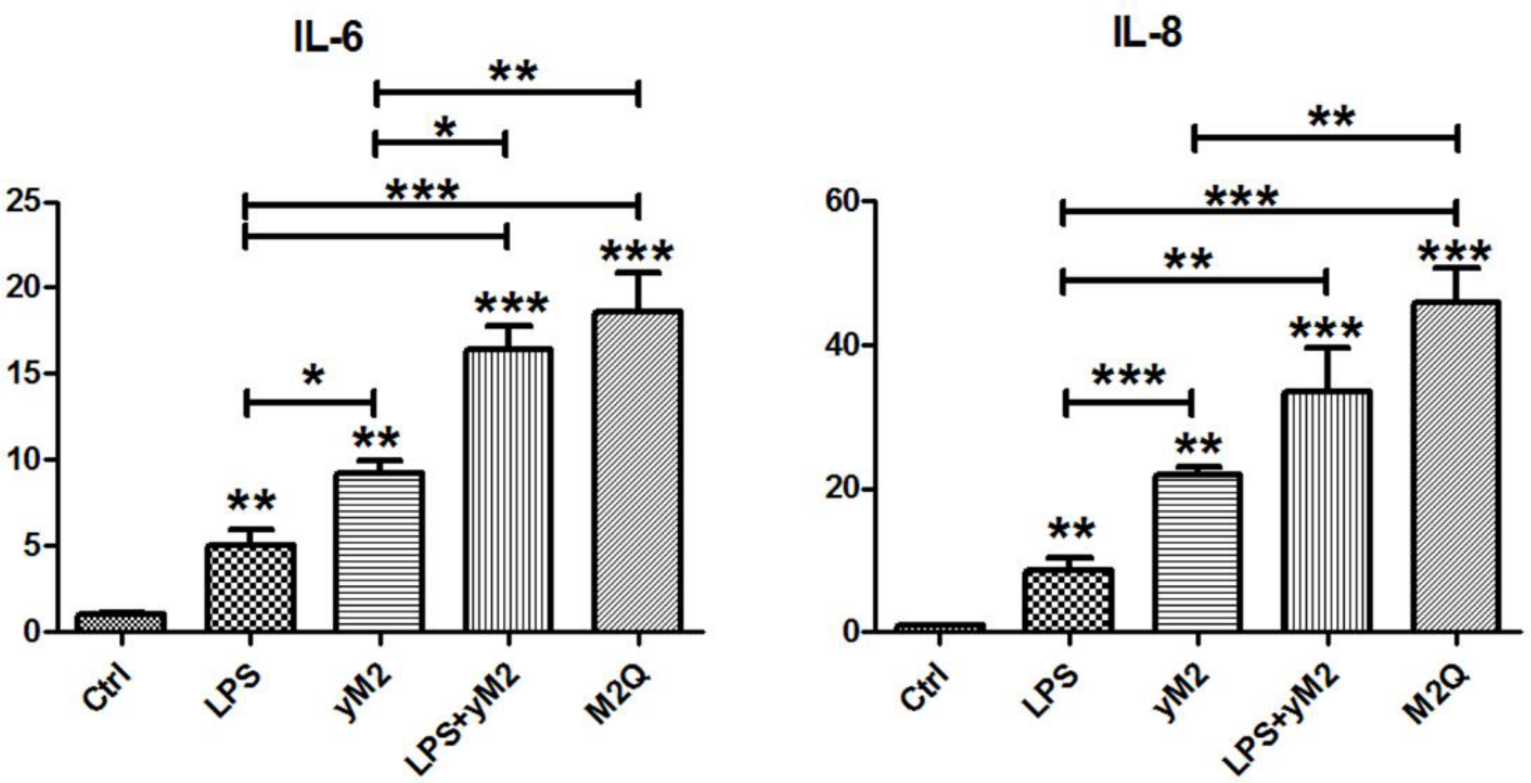
The effect of recombinant *Der m 2* protein on IL-6 and IL-8 mRNA expression in human BEAS-2B cells. The purified recombinant *Der m 2* (final 1 μg/ml) was added into the cell culture for 4 h of treatment, M2Q from *E. coli* and yM2 from yeast. The final concentration of LPS (0.8 ng/ml) that was equal to the endogenous LPS in M2Q was added into the cell culture for the same condition. Control was incubated for the same condition without adding anything. The total RNAs from cells were isolated by Tri reagent and reverse transcribed to cDNA before qPCR by specific primers. All assays were representative of at least three independent experiments performed in duplicate. ***P < 0.001, **P < 0.01, and *P < 0.05 compared with control or indicated pairs.

To verify the LPS-binding ability of M2Q, we performed cell-based experiments to investigate the allergic effects of M2Q. Because endogenous LPS present in M2Q can serve as an endotoxin, we performed the LAL assay to quantify the amount of endogenous LPS present in M2Q. In addition, we sensitized BEAS-2B cells, a human bronchial epithelial cell line, with the same concentration of LPS. Compared with the control, LPS induced approximately 5- and 8-fold IL-6 and IL-8 mRNA expression, respectively. However, M2Q with LPS induced considerably higher expression of IL-6 and IL-8 mRNA than did LPS alone in human BEAS-2B cells (**Figure 4**).

To confirm the effects of endogenous LPS, we expressed and purified another recombinant *Der m 2* from yeast (yM2). Compared with the control, yM2 induced 9- and 22-fold mRNA expression of IL-6 and IL-8, respectively (**Figure 4**). The findings significantly differed between LPS and yM2, indicating that *Der m 2* may exert stronger allergic effects. Furthermore, we treated cells with a combination of LPS and yM2 (LPS+yM2) and discovered that the effects of LPS+yM2 were similar to those of M2Q alone (**Figure 4**). The results suggested that LPS combined with recombinant *Der m 2* exerted a similar synergistic allergic effect; this finding is in accordance with those of previous studies (Yin et al., 2015; Radzyukevich et al., 2018).

### Asthma Animal–Based Model Established Using the Recombinant *Der m 2* Allergen

**Figure 5:**
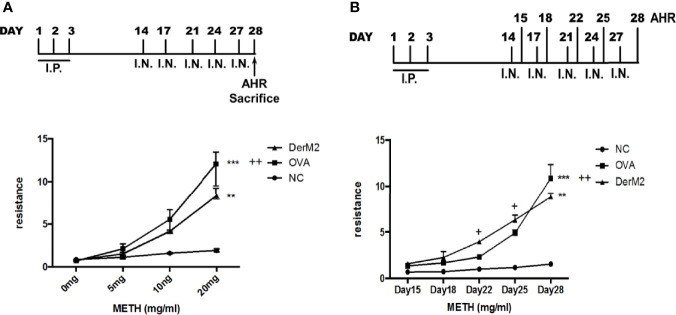
The effect of recombinant *Der m 2* protein on female BALB/c mice. **(A)** The upper panel is the mouse sensitization diagrammatic schedule. The mice were intraperitoneally injected with allergen or not in the first 3 days and were administered intranasal allergens at days 14, 17, 21, 24, and 27; total of 5 days. After the airway hyperactivity (AHR) determination, the mice were euthanized at day 28. The lower panel is the mouse’s AHR to methacholine (METH) assessed with the Buxco system. Normal group (NC) was shown as ●, *Der m 2* sensitization group was shown as ▲, ovalbumin (OVA) sensitization group was shown as ■. **(B)** The upper panel is the mouse sensitization diagrammatic schedule. The mice were treated as aforementioned but measured the AHR at days 15, 18, 22, 25, and 28. Normal group (NC) was shown as ●, *Der m 2* sensitization group was shown as ▲, OVA sensitization group was shown as ■. ***P < 0.001 and **P < 0.01 compared with NC; ^++^ *P* < 0.01 and ^+^ *P* < 0.05 compared with OVA.

*Der m* crude extracts caused asthma and airway inflammation in an animal model (Chang et al., 2015). In addition, *Der p 2* and *Der f 2* induce allergic asthma and inflammation in MD-2-deficient mice (Trompette et al., 2009; Ye et al., 2011) and in both C57BL/6 and BALB/c mice (Jiang et al., 2014; Choi et al., 2015), respectively. We investigated the allergic effect of *Der m 2*, a major component of *Der m* allergens, on sensitized mice. First, we sensitized mice with M2Q alone or OVA and then determined their AHR to various methacholine concentrations (**Figure 5A**). During the sensitization period, we observed that sensitized mice with M2Q alone developed asthma earlier. Therefore, we performed another sensitization experiment and determined the AHR of mice at days 15, 18, 22, 25, and 28. The result revealed that M2Q alone had induced more severe AHR in mice than did OVA at days 22 and 25 (**Figure 5B**). Although the final AHR of the M2Q-treated mice was weaker than that of the OVA-treated mice at day 28, the M2Q-induced AHR was still significantly greater than that of the control. These results suggested that the *Der m 2* component rapidly evoked moderate AHR in mice (**Figure 5**).

### FIP-*fve* Protein Restored Airway Hyperresponsiveness and Modulated the Mite Allergen-Specific Th1/Th2 Balance in *Der m 2*-Sensitized Mice

**Figure 6:**
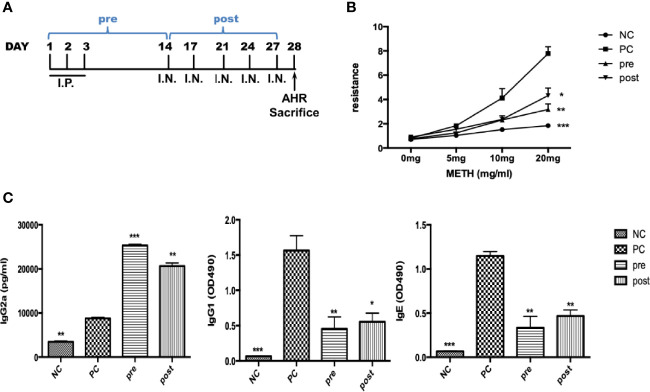
The effect of FIP-*fve* on AHR and immunoglobulins in recombinant *Der m 2*-sensitized BALB/c mice. **(A)** The draft of mouse sensitization schedule. The mice were intraperitoneally injected allergen or not in the first 3 days and were administered intranasal allergens at days 14, 17, 21, 24, and 27; total of 5 days. After the AHR determination, the mice were euthanized at day 28. The “pre” means the oral administration of FIP-*fve* once a day in the first 14 days, and “post” means the administration of FIP-*fve* in the last 14 days. **(B)** The mouse’s AHR to methacholine (METH) assessed with the Buxco system. Normal group (NC) was shown as ●, *Der m 2* (PC) sensitization group was shown as ■, pre group was shown as ▲, and post group was shown as ▼. **(C)** Allergen-specific IgE, allergen-specific IgG1, and allergen-specific IgG2a in sera were determined from the NC group, *Der m 2*-sensitized, and FIP-*fve* treatment (pre, post) mice. Results are representative of two independent experiments. ***P < 0.001, **P < 0.01, and *P < 0.05 compared with PC.

We established a *Der m 2*-induced airway inflammation and asthma mouse model (**Figure 6A**). We observed that pre- or post-cotreatment with FIP-*fve* significantly restored AHR at a methacholine dosage of 20 mg compared with no FIP-*fve* treatment in the mouse model (**Figure 6B**). In addition, similar immune responses relative to the traditional OVA-induced airway inflammation were executed for acute mouse asthma model in a previous study (Lee et al., 2013).

Th1/Th2 immune system balance tends to induce the production of allergen-specific IgG2a and IgE/IgG1. To examine immune responses, we analyzed the functional differentiation of allergen-specific immunoglobulins. Pretreatment and posttreatment with FIP-*fve* induced higher production of serum IgG2a antibodies compared with M2Q alone (PC; **Figure 6C**, left panel). Moreover, pretreatment and posttreatment with FIP-*fve* resulted in weaker immune responses of IgG1 and IgE antibodies than did M2Q alone (**Figure 6C**, middle and right panels). Our findings indicated that FIP-*fve* exerted immunomodulatory effects on mite allergen–specific Th2-skewed immune responses in *Der m 2*–sensitized mice (**Figure 6**).

### *Der m 2-*Induced Lung Inflammation and Airway Remodeling in Mice Could Be Alleviated by FIP-*fve* Protein

**Figure 7:**
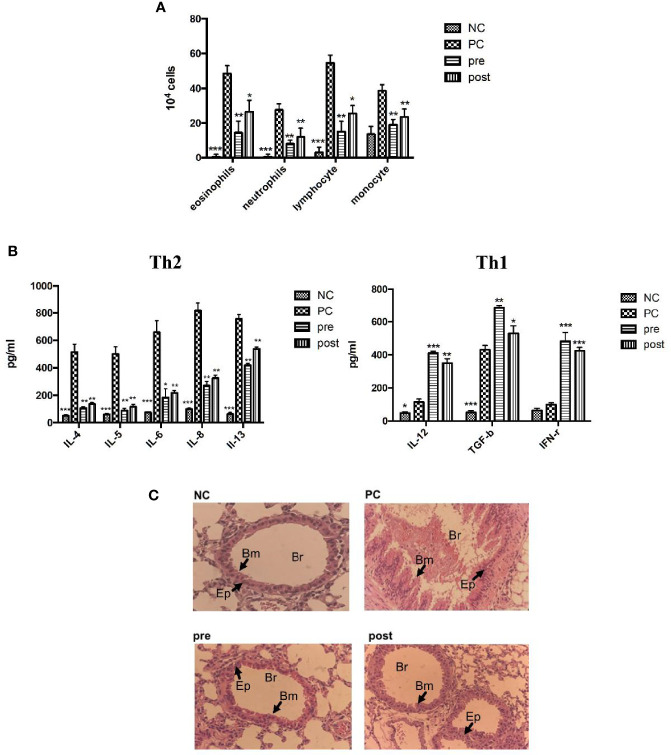
The effects of FIP-*fve* treatment on the infiltrating inflammatory cells and cytokines in the BALF and the airway inflammation of *Der m 2*-sensitized mice. **(A)** The inflammatory cells in the BALF of *Der m 2*-sensitized mice were shown, and the total cells and inflammatory cells were counted (×10^4^) from the BALF in millimeters by morphometric evaluations of cytospin preparations. **(B)** The different cytokine sets represent the Th1- and Th2-type responses. Th2-type cytokines were IL-4, IL-5, IL-6, IL-8, and IL-13 in the left panel, and Th1-type cytokines were IL-12, TGF-β, and IFN-γ in the right panel. ***P < 0.001, **P < 0.01, and *P < 0.05 compared with PC. **(C)** Histopathological image analysis on the airway inflammation in the lung tissue samples was obtained on day 28 at a magnification of ×100 using a light microscope. NC group, *Der m 2*-sensitized/challenged mice (PC), feeding in the first 14 days (pre), and in the last 14 days (post), respectively. All lung tissue was stained with hematoxylin and eosin on the sections to evaluate the inflammation severity and goblet cell hyperplasia (the arrows indicated region). Br, bronchus; Bm, basement membrane; Ep, epithelium.

IL-4, IL-5, and IL-13 are type 2 cytokines associated with asthma. Asthma is characterized by AHR, narrowing of the airway lumen diameter, and the infiltration of inflammatory cells. We evaluated whether *Der m 2* can induce asthma in mice. We examined three conditions of BALF in the lungs of *Der m 2*–sensitized mice that were not treated with FIP-*fve* to be positive control to evaluate the level of inflammatory cell infiltration and determine eosinophil, neutrophil, lymphocyte, and monocyte counts, which are the markers of airway inflammation. The mice pretreated or posttreated with FIP-*fve* had a lower level of inflammatory cell infiltration compared with the positive control mice (**Figure 7A**). To elucidate the involvement of cytokines, we examined immunological responses in the BALF of mice. We observed that IL-4, IL-5, IL-6, IL-8, and IL-13 were related to Th2-skewed immune responses, and IL-12, transforming growth factor (TGF)-β, and IFN-γ were associated with Th1-skewed immune responses. The levels of IL-4, IL-5, IL-6, IL-8, and IL-13 were increased in the *Der m 2*–treated mice. However, treatment with FIP-*fve* restored lung inflammation (pre and post; **Figure 7B**). The results revealed that FIP-*fve* protein ameliorated *Der m 2*–induced lung inflammation and Th1/Th2 cytokine imbalance in the BALF.

The histopathological imaging findings revealed that *Der m 2*–induced airway remodeling resulted in severe hyperplasia of airway basement membrane cells (**Figure 7C**, PC panel). Both pretreatment and posttreatment with FIP-*fve* alleviated lung inflammation in mice, and the hyperplasia of airway basement membrane cells was less severe in mice treated with FIP-*fve* than in those not treated with FIP-*fve* (**Figure 7C**, pre and post panels), similar to the normal group (**Figure 7C**, NC panel). These results were consistent with the aforementioned findings of allergen-specific IgG2a/IgG1/IgE. FIP-*fve* alleviated *Der m 2*–induced airway remodeling by modulating Th1-skewed cytokines and reducing the infiltration of inflammatory cells in BALF (**Figure 7**).

## Discussion

Group 2 allergens are one of the major allergens among mite allergens (Jin et al., 2003; Randall et al., 2005; Huang et al., 2020), and the allergenicity and IgE reactivity of the recombinant group 2 allergens are similar to those of their native allergens (Jin et al., 2003; Sookrung et al., 2016). Therefore, we examined the gene sequences of *Der m 2* and molecular functions of allergic *Der m 2* proteins. Moreover, we confirmed the sensitization ability of *Der m 2* in human bronchial epithelial cells and a BALB/c mouse model. *Der m 2* plays a crucial role in causing allergic asthma because it increased the cosensitization of *Der m* with other allergens (Huang et al., 2006).

LPS derived from Gram-negative bacteria can cause innate immune responses in human airway epithelial cells (Yin et al., 2015). However, recombinant *Der m 2* purified from yeast induced higher pro-inflammatory cytokines than LPS, and the combination of LPS and recombinant *Der m 2* obtained from yeast or *Der m 2* from *E. coli* and containing endogenous LPS induced the highest pro-inflammatory cytokines in the human airway epithelium (**Figure 4**). These findings indicated that the allergenicity of *Der m 2* is higher than that of LPS, and their combination exerts a synergistic effect (Radzyukevich et al., 2018), and this phenomenon may be caused by the homologous structure to MD-2 (**Figure 2**) that shares a similar function to directly interact with TLR4 complex (Trompette et al., 2009). Another possible route is that *Der p 2* stimulates the expression of MD-2 mRNA and protein, and then the recognition of LPS by MD-2 might reinforce the TLR4 signaling pathway (Trompette et al., 2009; Liao et al., 2015). In the acute phase, airway inflammation is mainly dominated by eosinophils (Pascoe et al., 2020; Wu et al., 2020) (**Figure 7A**). LPS abundance in HDMs altered the cytokine expression profile in the lung of mice. LPS inhibited Th2 cytokine production and increased IL-17 expression in mice (Daan de Boer et al., 2013; Zhao et al., 2017; Pascoe et al., 2020). Although LPS would enhance more severe inflammation in rats (Thakur et al., 2019), and in mouse the dosage of LPS would have influence on the phenotype of asthma (Kim et al., 2007). The low-dosage LPS (0.1 μg) with OVA caused eosinophil dominance in BALF, but the high-dosage LPS (>1 μg) with OVA caused neutrophil dominance in BALF. And the quantity of endogenous LPS in M2Q used for this animal study was less than 0.1 μg (<50 ng). In another study, Wang et al. (2021) used 50 μg crude *Der p* with 50 ng LPS to sensitize mice, and the results showed that LPS did not affect the level of HDM-induced infiltrated eosinophil and lymphocyte in mouse BALF. We suggested that the endogenous LPS in M2Q might have a slight effect on mice but did not affect the allergenicity of *Der m 2*, which had the LPS-binding ability.

FIP-*fve* exhibited 63% similarity to LZ-8, an FIP isolated from *Ganoderma lucidum*, and induced IFN-γ (Liu et al., 2012). FIP-*fve* induces IFN-γ through the p38 MAPK signaling pathway, calcium release, and protein kinase C-alpha activation in human PBMCs (Wang et al., 2004; Ou et al., 2009). FIP-*fve* alleviated inflammation induced by *Der m*, OVA, and RSV in the mouse airway (Lee et al., 2013; Chang et al., 2014; Chang et al., 2015). Plethysmography may not be an appropriate tool for evaluating airway resistance (Zhang et al., 2009). Thus, we confirmed the results by using allergen-specific antibodies in sera and by examining the presence of infiltrated cells and cytokines in the BALF. In this study, *Der m 2* induced Th2-skewed immune responses in mice, including allergen-specific IgE in serum (**Figure 6C**), eosinophil infiltration (**Figure 7A**), and Th2-skewed cytokine production (**Figure 7B**); these phenomena were similar to *Der p 2*- and *Der f 2*-induced immune responses (Lin et al., 2012; Stremnitzer et al., 2014). Besides the aforementioned IFN-γ induction by FIP-*fve* that could be observed in our study (**Figure 7B**), and the eosinophils were reduced (**Figure 7A**) by the inhibition of IL-5 (**Figure 7B**), which mediates the survival of eosinophils (Hsieh et al., 2007). In *Der m 2*-sensitized mice, FIP-*fve* reduced the Th2-related cytokines and elevated the Th1-related cytokines in mouse BALF (**Figure 7B**). And the effect of FIP-fve on cytokines also alleviated the inflammation in the mouse airway (**Figure 7C**) and mouse respiratory (**Figure 6B**). Therefore, FIP-*fve* protein can be a potential diet-based or pharmaceutical product that can alleviate HDM allergen-induced airway inflammation by exerting immunomodulatory effects.

To determine the potential characteristics of *Der m 2*, a major allergen of *Der m*, we systematically used *in silico* OMIC approaches, *in vivo* animal models, and *in vitro* translational medicine tools. In this study, we demonstrated the gene sequences of *Der m 2* and examined its molecular function and allergenicity. Our results revealed that FIP-*fve* can ameliorate allergic asthma caused by *Der m 2* by modulating airway inflammation.

## Data Availability Statement

The datasets presented in this study can be found in an online repository. The name of the repository and accession number can be found below: https://www.ncbi.nlm.nih.gov/protein/BDB45822.1.

## Ethics Statement

The animal study was reviewed and approved by Chung Shan Medical University Institutional Animal Care and Use Committee (Animal Experiment Approval number 2298).

## Author Contributions

K-HL, J-LK, and Y-FL were involved in the scheme or the division of labor. F-YY, T-SW worked on the *Der m 2-*specific mouse polyclonal antibodies. C-TW worked on the animal experiments. R-HH and Y-FL were involved in the NGS analysis, recombinant protein expression, and cell culture experiments. R-HH and C-TW drafted the article. K-HL, J-LK, and Y-FL discreetly revised the article. All authors contributed to the article and approved the submitted version.

## Funding

This work was supported by grants from the Ministry of Science and Technology (MOST 107-2320-B-040-010-MY3) and Chung Shan Medical University Hospital (CSH-2019-D-005, CSH-2020-D-002).

## Conflict of Interest

The authors declare that the research was conducted in the absence of any commercial or financial relationships that could be construed as a potential conflict of interest.

## Publisher’s Note

All claims expressed in this article are solely those of the authors and do not necessarily represent those of their affiliated organizations, or those of the publisher, the editors and the reviewers. Any product that may be evaluated in this article, or claim that may be made by its manufacturer, is not guaranteed or endorsed by the publisher.
