# Supplementary material for: Systematic Characterization of the Group 2 House Dust Mite Allergen in Dermatophagoides microceras
Source: Front Cell Infect Microbiol. 2022 Jan 17;11:793559. doi: 10.3389/fcimb.2021.793559 (PMC8801679; doi:10.3389/fcimb.2021.793559)
Supplement: Supplementary file 1 [file Image_1.pdf]

(A)

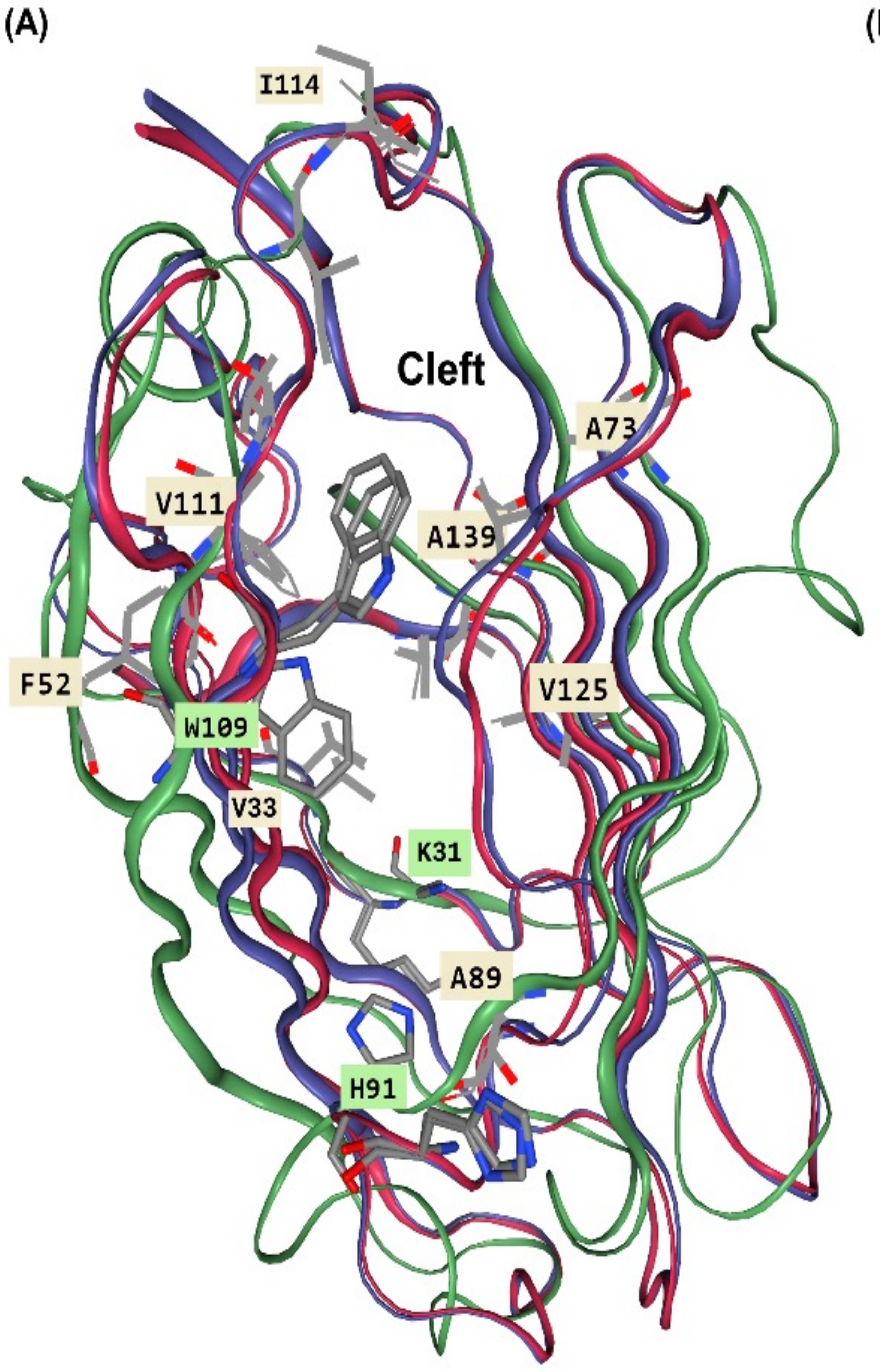


(B)

**Table | Summary the structural information used in this study.**

| Protein | Swiss-Protein Entry | PDB ID [a] | Method | Resolution (Å) | RMSD (Å) [b] |
|---|---|---|---|---|---|
| *Der f 2* | Q00855 | 1XWV | X-ray | 2.15 | 3.86 |
| *Der p 2* | P49278 | 1KTJ | X-ray | 1.83 | 3.91 |
| *Human MD2* | Q9Y6Y9 | 2E56 | X-ray | 2.0 | 2.95 |
| *Der m 2* | - | - | modelling | - | - |

a. The 3D structure of house dust mite group 2 allergens are retrieved from the RCSB Protein Database (http://www.rcsb.org/pdb/home/home.do). The color code is as follows: red, monomer A of *Der f 2* (remove ligand XPE and water); blue, monomer of *Der p 2* (remove water); green, homologous modelling structure *Der m 2* constructed by automated protein structure modeling with SwissModel server based on the template human LY96 (MD2) .

b. The two proteins should be superimposed using the "Magic fit" option relies on the correct detection of a stretch of similar residues at sequence level in the Swiss-PdbViewer tools and calculated the RMSD of $C\alpha$ atoms following global superposition of the model of *Der m 2* and the other target structures.

**Supplement Figure 1 | Comparison the 3D structures of the three HDMs major group 2 allergens. (A)** The superimposed main-chain backbone folds represented the individual monomers in the two crystals structures including *Der p 2* (color blue; PDB: 1KTJ) and *Der f 2* (color red; PDB: 1XWV), one additional homologous modelling structure *Der m 2* constructed by the human MD2 (color green; PDB: 2E56). This diagram was prepared with a browser-based NGL Viewer (http://nglviewer.org/ngl/). The predicted eight hydrophobic contacts and three hydrogen bond interactions of conserved residues to determine the amino acids contributing for ligand binding pocket are depicted white and green sticks labeled in a common orientation of **Fig.2C**, respectively. The hydrophobic cleft thought to server as a ligand binding site and sequence numbering convention is for the full-length protein of 146 residues of *Der m 2*. **(B)** The root-mean-square deviation (RMSD) between the human MD2 protein and group 2 allergen proteins of *Dermaphtophagoides* were estimated by SwissPdbViewer v.4.1.0 "Calculate RMS" option in "Fit" menu.

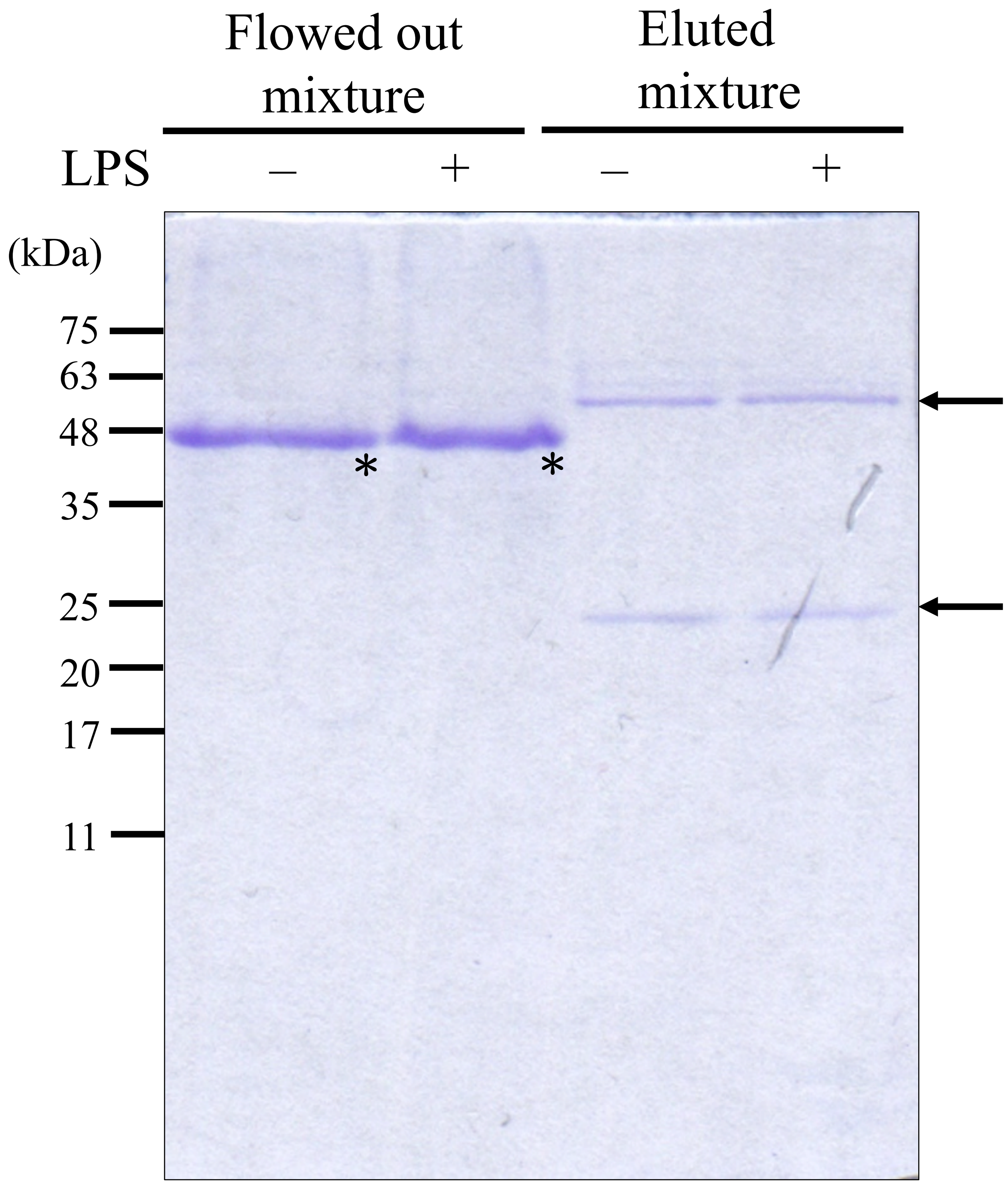


**Supplement Figure 2 | The CoIP result of OVA and LPS.**

The 100 μg OVA (~45 kDa) in the presence of 100 μg LPS or not were mixed with 5 μg anti-LPS antibody to precipitate LPS and its binding protein. The asterisks represent the OVA in the flowed out mixture, and the arrows represent the heavy chain and light chain of anti-LPS antibody in the eluted mixture.

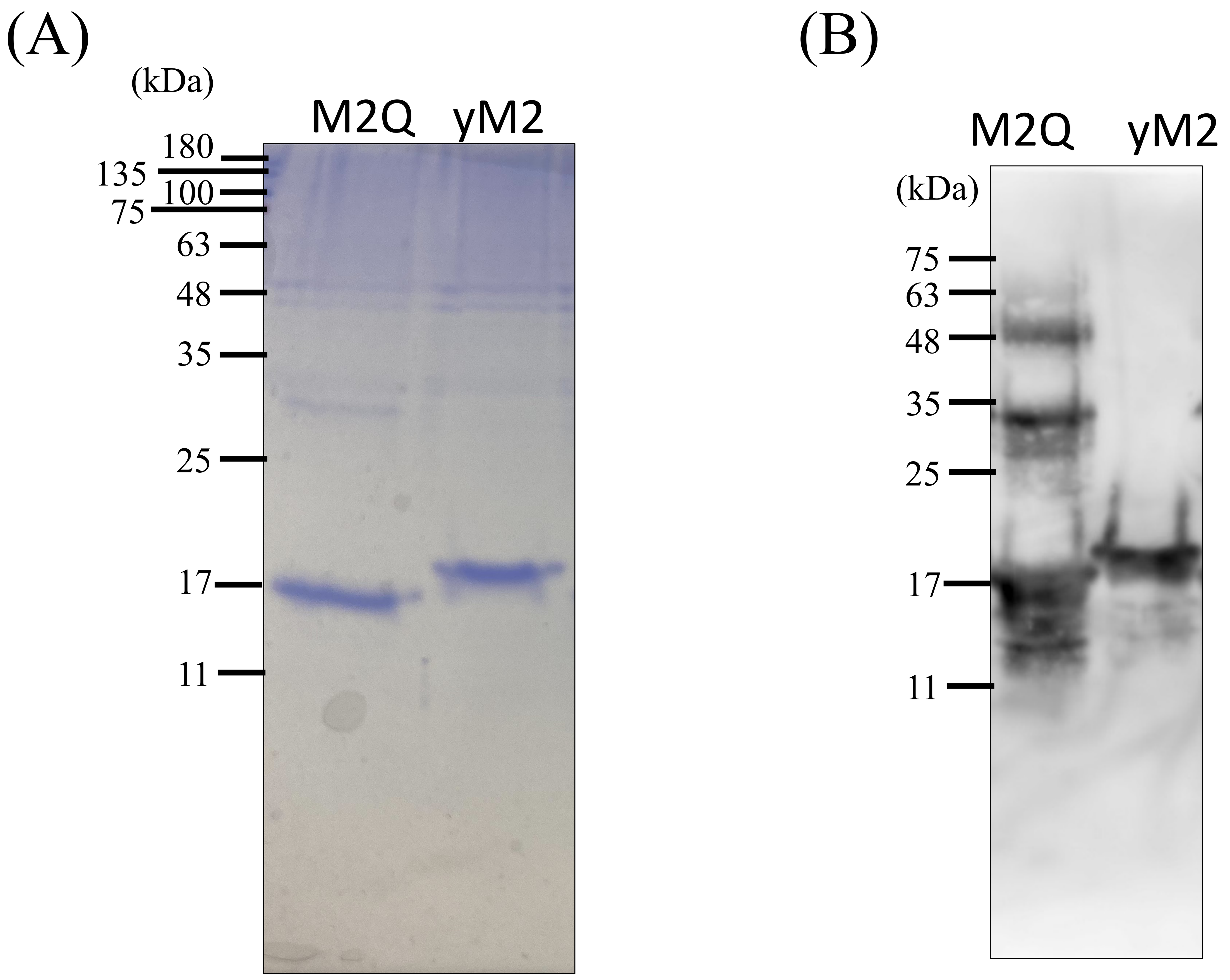


**Supplement Figure 3 | The purity of recombinant Der m 2.**
**(A)** The recombinant Der m 2 purified from E. coli (M2Q) and yeast (yM2) were loaded 4 μg for 15% SDS-PAGE. The molecular weight of M2Q and yM2 is approximate 15 kDa. **(B)** The result of Western blot for the recombinant Der m 2 by using mouse anti-Der m 2 antibody and goat anti-mouse HRP antibody.
